# Adverse childhood experiences and cognitive function in later life: the sequential mediating roles of education level and adult loneliness

**DOI:** 10.3389/fpubh.2024.1409966

**Published:** 2024-07-16

**Authors:** Xiaojuan Deng, Min Xie, Yu Wang, Jia Cai, Min Zou, Qiang Wang

**Affiliations:** ^1^Mental Health Center, West China Hospital, Sichuan University, Chengdu, Sichuan, China; ^2^Sichuan Clinical Medical Research Center for Mental Disorders, Chengdu, China; ^3^Department of Pharmacy, West China Hospital, Sichuan University, Chengdu, China

**Keywords:** adverse childhood experiences, education attainment, loneliness, cognitive function, older adult, Chinese

## Abstract

**Background:**

This study assesses the impact of adverse childhood experiences (ACEs) on the cognitive function of older adults. Furthermore, it examines the potential underlying mechanism involving education level and the subjective “feeling of loneliness” (FOL).

**Methods:**

Analyzing a population-based cohort sample from the China Health and Retirement Longitudinal Study database, 8,365 subjects aged 45 or older were interviewed in 2018. Ten ACEs indicators were measured using life history questionnaires assessed at 2014. FOL was assessed using a single item from 10-item Center for Epidemiological Studies Depression Scale (CESD-10). Cognitive function was assessed using a structured questionnaire comprising four dimensions: memory, orientation, computation, and visuospatial abilities.

**Results:**

In the fully adjusted model, which accounted for age, gender, marital status, smoke, drink, rural residence, and education levels of both mothers and fathers, the linear regression analysis indicated that ACEs were inversely associated the lower education level (B = −0.058, 95% CI = −0.090, −0.026, *p* < 0.001), and ACEs were found to be linked to an elevated risk of FOL (B = 0.072, 95% CI = 0.056, 0.089, *p* < 0.001). In addition, ACEs was not significantly associated with cognitive function (B = −0.047, 95% CI = −0.108, 0.015, *p* = 0.136), but FOL was significantly associated with cognitive function (B = −0.483, 95% CI = −0.561, −0.404, *p* < 0.001). Mediation analysis revealed that education level and FOL sequentially and partially mediated the association between ACEs and the total cognitive score, with a proportion mediated of 52.58%.

**Limitations:**

The evaluation of ACEs exposure was based on binary response options. This method limited our ability to explore various dimensions of adversity, such as ages of occurrence, severity, frequency, duration, and the extent of psychological effects at the time. Furthermore, the assessment of loneliness relied on a single item from the CESD-10, introducing a potential source of measurement error.

**Conclusion:**

Our study unveils a substantial association between ACEs and education level, as well as with FOL and cognitive function in the older adults. Moreover, education level and FOL serve as sequential mediating factors in the relationship between ACEs and cognitive function.

## 1 Introduction

Adverse childhood experiences (ACEs) refer to stressful or traumatic events encountered during childhood, specifically defined as incidents occurring before the age of 18 (1). Over 60% of adults report experiencing at least one adverse childhood experience, and 17% report four or more ACEs. ACEs persistently impact adverse health outcomes throughout an individual’s life, contributing to lower educational attainment, significant causes of adult mortality and influencing various psychosocial outcomes linked to mental illness and poor health, as well as substantial financial costs (2–6).

In China, the escalating aging population has brought about heightened concerns regarding cognitive decline (7). This decline among the older adults can lead to various adverse outcomes, including health complications, reduced social interaction, heightened safety hazards, greater medical complexities, and financial strains (8). Studies have indicated a connection between ACEs and cognitive impairment in adulthood, particularly impacting memory, processing speed, and executive functions, even in later life stages (9–13). Further investigations into the enduring cognitive consequences of specific ACEs could shed light on the specificity and pathways linking individual ACEs to cognitive functions in later life. Some studies have adopted a mediation framework to explore these associations. By examining these potential pathways, invaluable insights could be gained to develop more effective intervention and prevention strategies aimed at mitigating the risks associated with cognitive impairment.

Numerous studies have already found that individuals who have experienced ACEs are more likely to have lower educational attainment (6, 14). This early exposure to ACEs has been linked to a decline in educational achievement, contributing to variations in cognitive skills that manifest in early adulthood and persist into older age, thereby increasing the risk of age-associated cognitive decline and late-life dementia (15). Moreover, recent research has identified specific adverse experiences that may further exacerbate cognitive declines by influencing educational attainment. For instance, one study demonstrated a robust and enduring indirect association between parental substance abuse and poorer cognitive function in later life, mediated through educational level (16). Similarly, Halpin et al. observed that heightened childhood adversity could amplify susceptibility to cognitive impairment by shaping the early educational experiences of older adults (17). Additionally, Burr et al. found that adult loneliness partially mediated the relationship between childhood friendship experiences and cognitive function in later life (18). These findings collectively underscore the intricate relationship between ACEs, educational level, and cognitive function across the lifespan. They highlight the importance of considering the long-term consequences of ACEs on educational outcomes and cognitive health in later life.

Loneliness, characterized as the perception of social isolation or the subjective feeling of being alone (19), represents a significant public health concern that often goes unrecognized or trivialized (20). Considering the escalating number of older adults, it becomes crucial to acknowledge the detrimental effects of loneliness, as revealed in both animal models and longitudinal human investigations (21–23). Substantial studies have found that loneliness was associated with objective social isolation, depression, or poor social skills, cognitive impairment and premature mortality (23–25). Emerging evidence have suggested that those who have been exposed to ACEs are more likely to have the feeling of loneliness (26, 27). For example, individuals who are exposed to ACEs report low self-esteem and problems with emotional regulation, which may lead to encounter challenges in experiencing pleasure, contentment, and happiness. Lin et al. found that ACEs were associated with feeling of loneliness (FOL) trajectory, which play a mediating role between ACEs and problematic internet use in young adults (28). Besides, the mediating role of loneliness have been identified between ACEs and pain, problematic internet abuse and depressive symptoms (28–30). Therefore, one potential explanation for the link between ACEs and cognitive impairment is exposure to adverse events in early life, which can lead to increased feelings of loneliness (FOL) later in life, thus resulting in greater impairment of cognition. However, there remains a scarcity of research delving into the precise mechanisms through which ACEs elevate the risk of cognitive deficits, including factors like educational level and subjective FOL. Exploring these potential pathways could provide valuable insights for the development of more effective intervention and prevention strategies aimed at mitigating the risks associated with cognitive impairment.

There is a lack of studies examining the association between ACEs and the decline of cognitive functioning in later life through a sequential mediating pathway. There could exist a direct relationship between ACEs and cognition. Alternatively, depending on the strength of the mediators, there may be an indirect pathway involving educational level and FOL. Through these indirect pathways, it is possible to elucidate the mechanisms through which ACEs contribute to an elevated risk of cognitive impairment. Drawing from the literature discussed earlier, it can be inferred that the cumulative effect of ACEs may increase the likelihood of lower cognitive function among older individuals. Consequently, we have formulated four hypotheses to guide our investigation:

*H1:* ACEs are inversely associated with cognitive function among the older adults.

*H2:* Educational level serves as a mediator in the relationship between ACEs and cognitive function among the older adults.

*H3:* FOLs acts as a mediator in the relationship between ACEs and cognitive function among the older adults.

*H4:* Educational level and FOLs sequentially mediate the relationship between ACEs and cognitive function among the older adults.

**Figure 1:**
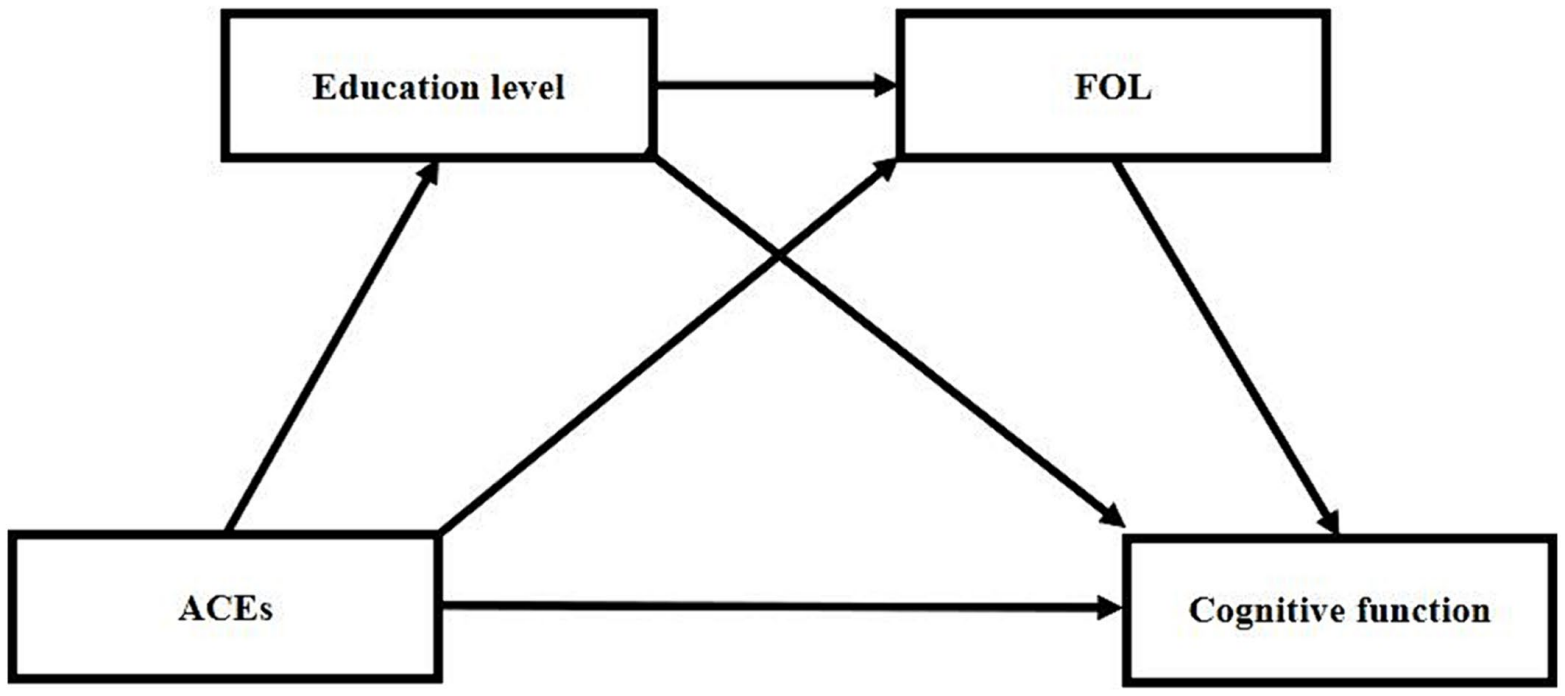
Hypothesized model of ACEs, education level, FOLs and cognitive function. ACEs, adverse childhood experiences; FOL, feeling of lonely.

Figure 1 illustrates the proposed research model for the current study.

Therefore, leveraging the unique longitudinal cohort of the China Health and Retirement Longitudinal Study (CHRLS), the current aimed to (1) examine the association between ACEs and education level, FOL and cognitive function in older adults; (2) investigate the connection between FOL and cognitive function; and (3) determine if education level and FOL sequentially mediated the association between ACEs and cognitive function.

## 2 Materials and methods

### 2.1 Study design and participants

This study utilized data from fourth wave (2018) and Life History Survey Questionnaire data (2014) obtained from a nationally representative longitudinal survey (CHARLS), which conducted by the China Center for Economic Research at Peking University. Eligible participants, aged 45 and above, were enrolled using a multistage probability sampling method, with follow-up interviews conducted every 2 years. The datasets are accessible for download on the CHARLS homepage at http://charls.pku.edu.cn/en. Approval for the CHARLS survey project was granted by the Biomedical Ethics Committee of Peking University, and all participants were required to provide informed consent through signing.

### 2.2 Data collection instruments

#### 2.2.1 Demographic information

The general information included age, gender, education level, married status, smoke, drink, rural residence and education levels of both mothers and fathers (Table 1; Supplementary Table S1). The married status is a binary variable representing either “married” or “not in marriage.” Smoking and drinking are binary variables that signify the presence or absence of a history of smoking and alcohol consumption. Rural residence is also a binary variable indicating whether individuals live in urban or rural areas. The education level includes: no formal education illiterate (1), did not finish primary school but cap (2), sishu (a type of educational institution in China that focuses on the early education and development of young children) (3), elementary school (4), middle school (5), high school (6), vocational school (7), two/three-year college/associate degree (8), four-year college/bachelor’s degree (9), and post-graduated (master/PhD) (10). And the marry status includes: married, partnered, separated, divorced, widowed, and never married. In our study, we utilized rural residence, and the educational levels of both mothers and fathers as covariates. These factors have been previously linked to cognitive function and were included to account for their potential influence on our findings.

**Table 1:** The demographic characteristics of participants assessed at 2018 (*n* = 8,365).

	Mean ± SD/*n* (%)	Range
Age, year	58.83 ± 8.87	
Gender		
Male	3,824 (45.71%)	
Female	4,541 (54.29%)	
Education level		
No formal education illiterate	1,489 (17.80%)	
Did not finish primary school but cap	1,139 (13.62%)	
Sishu	9 (0.11%)	
Elementary school	2,777 (33.20%)	
Middle school	1,850 (22.12%)	
High school	746 (8.92%)	
Vocational school	188 (2.25%)	
Two/Three Year College/Associate degree	119 (1.42%)	
Four Year College/Bachelor’s degree	44 (0.53%)	
Post-graduated (Master/PhD)	4 (0.05%)	
Marital status		
Married	7,827 (93.57%)	
Not in marriage	538 (6.43%)	
Smoke		
Yes	3,912 (46.77%)	
No	4,453 (53.23%)	
Drink		
Yes	4,380 (52.36%)	
No	3,985 (47.64%)	
Residence		
Urban	3,541 (42.33%)	
Rural	4,824 (57.67%)	
Total cognitive scores	12.04 ± 3.56	0–20
Memory	4.48 ± 1.96	0–10
Orientation	3.21 ± 0.91	0–4
Computation	3.66 ± 1.49	0–5
Visuospatial abilities	0.68 ± 0.46	0–1
Subjective feeling of lonely		
Rarely or none	6,184 (73.93%)	
Some or a little	1,085 (12.97%)	
Occasionally or a moderate	636 (7.60%)	
Most or all	460 (5.50%)	
Number of ACEs		
0	167 (2.00%)	
1	2,886 (34.50%)	
2	2,944 (35.19%)	
3	1,541 (18.42%)	
> = 4	827 (9.89%)	

ACEs, Adverse childhood experiences; SD, standard error.

#### 2.2.2 Adverse childhood experiences

The ACEs included ten types of adverse experiences occurred during childhood, including emotional neglect, physical abuse, domestic violence, unsafe neighborhood, and bullying, parental separation or divorce, parental death, household substance abuse, household mental illness, and incarcerated household member (31). The ACEs were quantified as cumulative scores, ranging from 0 to 10, and subsequently classified into four groups: 0 and 1, 2, 3, and 4 or more.

#### 2.2.3 Loneliness symptom

A solitary item from the 10-item Center for Epidemiological Studies Depression Scale (CESD-10) was employed to evaluate loneliness symptoms throughout investigation conducted in 2018. Respondents were instructed to assess the frequency of feeling lonely during the past week, assigning scores within the range of 0 to 3. The survey utilizes a 4-point Likert scale: 0 indicating very rarely or not at all (< 1 day), 1 representing not too much (1–2 days), 2 signifying occasionally or about half the time (3–4 days), and 3 denoting most of the time (5–7 days).

#### 2.2.4 Cognition

This research, conducted using data from the fourth wave assessment of cognitive function, focused on four dimensions: episodic memory, orientation, computation, and visuospatial abilities. Episodic memory was assessed through immediate and delayed word recall, with participants recalling as many words as possible after hearing 10 Chinese nouns, both immediately and 4–10 min later. The episodic memory score was determined as the average of immediate and delayed word recalls, ranging from 0 to 10. Orientation and computation were measured using the 10 items in the Telephone Interview for Cognitive Status (TICS-10), rated on a scale of 0 to 10. Orientation involved inquiries about the date (month, day, year), day of the week, and season. Computation was evaluated by subtracting 7 from 100 five times consecutively, resulting in scores ranging from 0 to 5. Visuospatial abilities were evaluated through graphic rendering, where respondents were presented with a painting and asked to draw a similar figure. The scoring range for drawing is from 0 to 1, with successful drawings earning 1 point and unsuccessful attempts receiving 0 points. The cognitive score, comprising TICS-10, word recall, and graph drawing, ranged from 0 to 21, with higher scores indicating better cognitive function.

### 2.3 Statistical analysis

Linear regression analyses were conducted to investigate the relationships between ACEs and outcomes of education level and FOL. In Model 1, ACEs were included; in Model 2, covariates such as age, gender, smoke, drink, marital status, rural living or education level were added to Model 1; in Model 3, education level of mother and father were added to Model 2. Furthermore, linear regression analyses were carried out to explore the association between ACEs and cognitive function. In Model 1, ACEs were included; in Model 2, FOL was added into Model 1; in Model 3, covariates such as age, gender, smoke, drink, marital status, rural living or education level were added to Model 3; in Model 4, education level of mother and father were added to Model 3. Spearman correlation analysis was conducted to examine the relationship between ACEs, education level, FOL, and total cognitive score, with Bonferroni correction applied for multiple comparisons. Finally, a serial mediation analysis was used to examine the sequential mediated effects of education level and FOL on the relationship between ACEs and cognitive function (i.e., ACEs (X), education level (M1), FOL trajectories (M2) and total cognitive score (Y)). The significant level of estimates was set as two-tailed *p* < 0.05. Serial mediation analysis was conducted by MPLUS (version 8.3) and other statistical analyses were conducted using Stata (version 17.1).

## 3 Results

### 3.1 Participant characteristics

The demographic, ACEs, loneliness scores and cognition of the 8,365 selected subjects are described in Table 1. The 8,365 participants included 3,824 (45.71%) male and 4,541 (54.29%) female, with mean age of 58.83 ± 8.87. 93.57% of participants are married, while 6.43% are not in a marital relationship. There are 3,912 (46.77%), 4,380 (52.36%) and 4,824 (57.67%) participants reported smoke, drink and living in rural, respectively. The education levels of the subjects are also detailed in Table 1, and the education levels of their mothers and fathers are detailed in Supplementary Table S1. The total cognitive score, memory, orientation, computation and drawing scores assessed at wave 4 were 12.04 ± 3.56 (range: 0–20), 4.48 ± 1.96 (range: 0–10), 3.21 ± 0.91 (range: 0–4), 3.66 ± 1.49 (range: 0–5) and 0.68 ± 0.46 (range: 0–1), respectively. There are 6,184 (73.93%), 1,085 (12.97%), 636 (7.60%) and 460 (5.50%) subjects reported very rarely or not at all (< 1 day), not too much (1–2 days), occasionally or about half the time (3–4 days), and most of the time of FOL, respectively. And there are 167 (2.00%), 2,886 (34.50%), 2,944 (35.19%), 1,541 (18.42%) and 827 (9.89%) reported 0, 1, 2, 3, 4 or above adverse experiences.

### 3.2 Association between ACEs and education level

The linear regression analysis showed that ACEs were associated with lower education level (B = −0.078, 95% CI = −0.113, −0.042, *p* < 0.001) in Model 1 (Table 2), without adjusting for covariates. In Model 2, after adjusting for age, gender, marital status, smoke, drink, and rural residency, ACEs remained significantly associated with education level (B = −0.066, 95% CI = −0.098, −0.033, *p* < 0.001). In Model 3, after further adjustment for the education levels of both the mothers and fathers, ACEs remained significantly associated with education level (B = −0.058, 95% CI = −0.090, −0.026, *p* < 0.001).

**Table 2:** Association between ACEs and education level.

	Model 1			Model 2			Model 3		
	B	95% CI	*p*	B	95% CI	*p*	B	95% CI	*p*
ACEs	−0.078	−0.113, −0.042	<0.001	−0.066	−0.098, −0.033	<0.001	−0.058	−0.09, −0.026	<0.001
Age	–	–	–	−0.030	−0.034, −0.026	<0.001	−0.019	−0.023, −0.014	<0.001
Gender	–	–	–	−0.909	−1.021, −0.797	<0.001	−0.939	−1.048, −0.83	<0.001
Marital status				0.000	−0.072, 0.072	1.000	0.005	−0.065, 0.076	0.883
Smoke				−0.172	−0.276, −0.067	<0.001	−0.150	−0.252, −0.048	0.004
Drink				0.126	0.042, 0.210	0.003	0.115	0.033, 0.196	0.006
Rural living	–	–	–	−0.831	−0.902, −0.759	<0.001	−0.699	−0.77, −0.628	<0.001
Father’s education level	–	–	–	–	–	–	0.161	0.138, 0.183	<0.001
Mother’s education level	–	–	–	–	–	–	0.114	0.081, 0.146	<0.001

ACEs, adverse childhood experiences; B, non-standard regression coefficient; CI, confidence interval.Model 1: added ACEs; Model 2: added age, gender, marital status, smoke, drink and rural living into model 1; Model 3: added education levels of both mothers and fathers to model 2.

### 3.3 Association between ACEs and FOL

The linear regression analysis revealed that ACEs were linked to an increased risk of FOL (B = 0.074, 95% CI = 0.057, 0.091, *p* < 0.001) in Model 1 (Table 3), prior to adjusting for any covariates. In Model 2, even after accounting for factors such as age, gender, education level, marital status, smoke, drink and rural residency, the association between ACEs and an elevated risk of FOL remained significant (B = 0.072, 95% CI = 0.056, 0.089, *p* < 0.001). Further adjustments in Model 3, which included the educational levels of both parents, still showed a significant association between ACEs and an increased risk of FOL (B = 0.072, 95% CI = 0.056, 0.089, *p* < 0.001).

**Table 3:** Association between ACEs and FOL.

	Model 1			Model 2			Model 3		
	B	95% CI	*p*	B	95% CI	*p*	B	95% CI	*p*
ACEs	0.074	0.057, 0.091	<0.001	0.072	0.056, 0.089	<0.001	0.072	0.056, 0.089	<0.001
Age	–	–	–	0.001	−0.002, 0.003	0.568	0.000	−0.002, 0.002	0.813
Gender	–	–	–	−0.233	−0.292, −0.175	<0.001	−0.231	−0.289, −0.173	<0.001
Education level	–	–	–	−0.024	−0.035, −0.013	<0.001	−0.022	−0.034, −0.011	<0.001
Marital status				0.143	0.106, 0.180	<0.001	0.143	0.106, 0.180	<0.001
Smoke				0.108	0.054, 0.161	<0.001	0.107	0.054, 0.161	<0.001
Drink				0.008	−0.035, 0.051	0.710	0.008	−0.035, 0.051	0.702
Rural living	–	–	–	0.102	0.065, 0.140	<0.001	0.099	0.061, 0.137	<0.001
Father’s education level	–	–	–	–	–	–	−0.007	−0.019, 0.005	0.239
Mother’s education level	–	–	–	–	–	–	−0.002	−0.019, 0.015	0.787

ACEs, adverse childhood experiences; B, non-standard regression coefficient; CI, confidence interval.Model 1: added ACEs; Model 2: added age, gender, education level, marital status, smoke, drink and rural living into model 2; Model 3: added education levels of both mothers and fathers to model 2.

### 3.4 Association between ACEs, FOL, and cognition

The linear regression analysis revealed a significant association between ACEs and poor cognitive function (B = −0.082, 95% CI = −0.148, −0.015, *p* = 0.016). However, in Model 2, after incorporating FOL into Model 1, ACEs lost their significant association with cognition (B = −0.038, 95% CI = −0.104, 0.028, *p* = 0.258), while FOL displayed a significant inverse association with cognition (B = −0.589, 95% CI = −0.673, −0.505, *p* < 0.001). Advancing to Model 3, which integrated covariates such as age, gender, education level, marital status, smoke, drink, and rural residency into Model 2, ACEs were not associated with cognition (B = −0.050, 95% CI = −0.111, 0.012, *p* = 0.122), while FOL retained its significant association with cognition (B = −0.485, 95% CI = −0.564, −0.407, *p* < 0.001). Transitioning to Model 4, with the additional inclusion of the education levels of both mother and father into Model 3, FOL maintained a significant association with poor cognitive function (B = −0.483, 95% CI = −0.561, −0.404, *p* < 0.001). However, ACEs no longer exhibited an association with cognitive function (B = −0.047, 95% CI = −0.108, 0.015, *p* = 0.136, refer to Table 4).

**Table 4:** Association between ACEs, FOL, and cognition.

	Model 1			Model 2			Model 3			Model 4		
	B	95% CI	*p*	B	95% CI	*p*	B	95% CI	*p*	B	95% CI	*p*
Number of ACEs	−0.082	−0.148, −0.015	0.016	−0.038	−0.104, 0.028	0.258	−0.050	−0.111, 0.012	0.122	−0.047	−0.108, 0.015	0.136
FOL	–	–	–	−0.589	−0.673, −0.505	<0.001	−0.485	−0.564, −0.407	<0.001	−0.483	−0.561, −0.404	<0.001
Age	–	–	–	–	–	–	−0.105	−0.113, −0.097	<0.001	−0.099	−0.107, −0.091	<0.001
Gender	–	–	–	–	–	–	0.976	0.763, 1.190	<0.001	0.943	0.729, 1.156	<0.001
Education level	–	–	–	–	–	–	0.283	0.243, 0.323	<0.001	0.261	0.220, 0.302	<0.001
Marital status							−0.085	−0.221, 0.051	0.222	−0.080	−0.216, 0.056	0.247
Smoke							−0.474	−0.67, −0.278	<0.001	−0.469	−0.665, −0.274	<0.001
Drink							0.170	0.013, 0.327	0.033	0.168	0.011, 0.325	0.036
Rural living	–	–	–	–	–	–	−1.024	−1.163, −0.886	<0.001	−0.976	−1.115, −0.836	<0.001
Father’s education level	–	–	–	–	–	–	–	–	–	0.044	0.000, 0.088	0.048
Mother’s education level	–	–	–	–	–	–	–	–	–	0.119	0.056, 0.181	<0.001

ACEs, adverse childhood experiences; B, non-standard regression coefficient; CI, confidence interval; FOL, feeling of loneliness.Model 1: added ACEs; Model 2: added FOL to Model 1; Model 3: added age, gender, education level, marital status, smoke, drink and rural living into model 2; Model 4: added education level of both mothers and fathers to model 3.

### 3.5 Spearman relationship analysis of variables of interests

The Spearman relationship analysis revealed inversely correlations between ACEs and educational level (r = −0.048, *p* < 0.001) as well as cognitive function (r = −0.026, *p* = 0.016). ACEs exhibited a positive relationship with FOL (r = 0.076, *p* < 0.001). Educational level demonstrated an inversely correlation with FOL (r = −0.038, *p* < 0.001) and a positive association with cognitive function (r = 0.200, *p* < 0.001). FOL was inversely associated with cognitive function (r = −0.126, *p* < 0.001). The association between ACEs and cognitive function was failed to undergo Bonferroni correction (r = −0.026, *p* = 0.098). The correlation analysis of other variables of interest has undergone multiple corrections, with all *p*-values remaining below 0.01 after Bonferroni correction (Table 5).

**Table 5:** Spearman relationship between number of ACEs, education level, FOL, and cognition.

	ACEs	Education level	FOL	Total cognitive scores
ACEs	1.000			
Education level	−0.048 ***	1.000		
FOL	0.076 ***	−0.038 ***	1.000	
Total cognitive scores	−0.026 *	0.200 ***	−0.126 ***	1.000

ACEs, adverse childhood experiences; FOL, feeling of lonely.*: *p* < 0.05; ***: *p* < 0.001.

### 3.6 Chain mediation analysis

**Figure 2:**
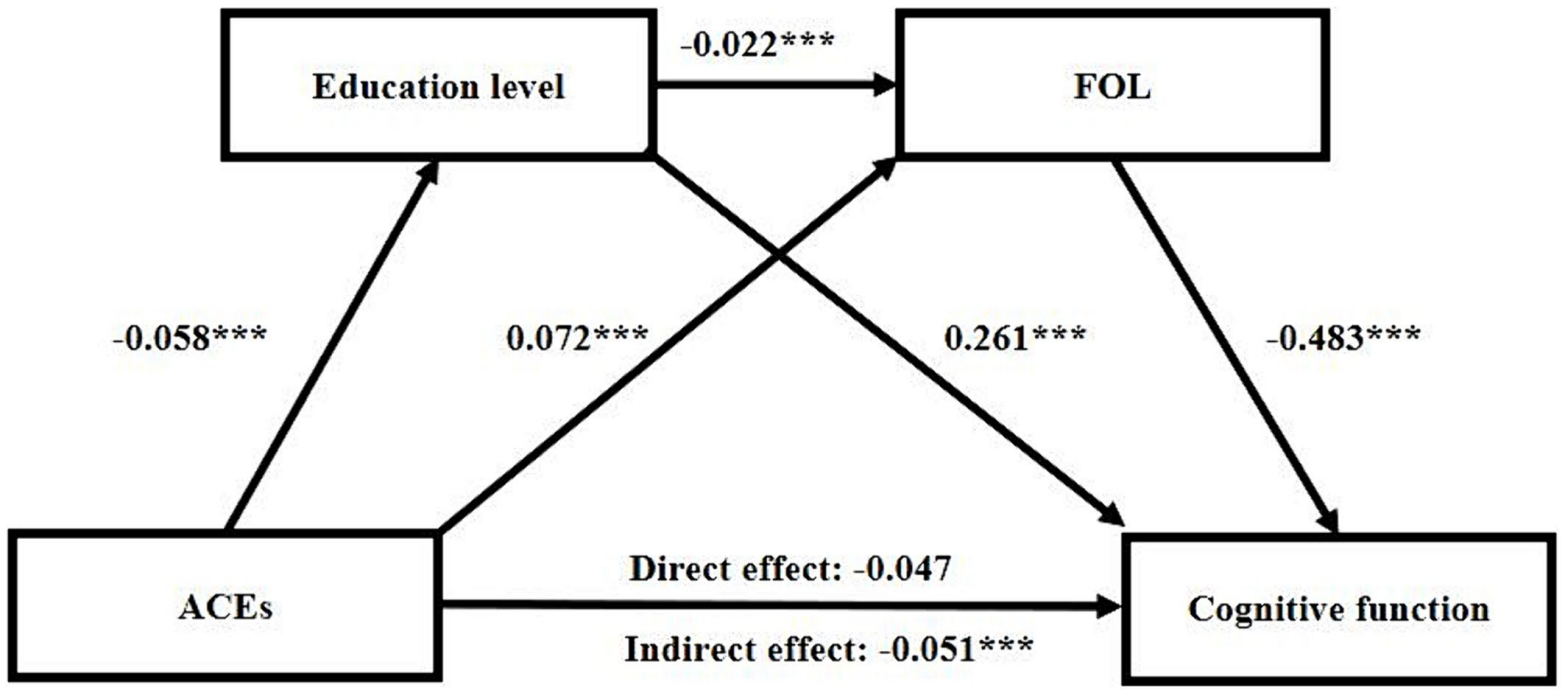
Chain mediation analysis between ACEs and cognition. The models utilize the ACEs path, as indicated in the 2014 Life History Survey Questionnaire assessments, as the independent variable. The sequential mediators are education level and FOL, while the dependent variable is the total cognition score assessed at wave 4. The mediation model is adjusted for age, gender, rural living and education level of mothers and fathers. ACEs, adverse childhood experiences; FOL, feeling of lonely. *p < 0.05; **p < 0.01; ***p < 0.001.

Figure 2 illustrates the sequential mediating roles of educational level and FOL between ACEs and cognitive function. In the initial model, we tested the association between ACEs and educational level. The results (*R*^2^ = 0.206, *F* = 26.826, *p* < 0.001) revealed a positive relationship, indicating that ACEs were inversely correlated with educational level (B = −0.058, 95% CI = −0.086, −0.031, *p* < 0.001). Subsequently, the model examined whether ACEs and educational level were directly linked to the FOL. The outcomes (*R*^2^ = 0.031, *F* = 7.786, *p* < 0.001) demonstrated a positive correlation between ACEs and FOL (B = 0.072, 95% CI = 0.057, 0.087, *p* < 0.001), while educational level exhibited a negative association with FOL (B = −0.022, 95% CI = −0.032, −0.013, *p* < 0.001). Finally, we explored the relationship among ACEs, education level, FOL, and cognitive function. The results (*R*^2^ = 0.166, *F* = 22.734, *p* < 0.001) indicated a direct and positive association between educational level and cognitive function (B = 0.261, 95% CI = 0.226, 0.296, *p* < 0.001). FOL was inversely related to cognitive function (B = −0.483, 95% CI = −0.551, −0.416, *p* < 0.001). However, the association between ACEs and cognitive function was not significant (B = −0.047, 95% CI = −0.096, 0.005, *p* = 0.136) (see Supplementary Table S2). We employed the bootstrap method with 5,000 iterations to conduct the mediating effect test. The mediating effect of educational level and the sequential mediating effects of FOL were considered significant if the 95% confidence interval did not include 0. The total indirect effect of ACEs (B = −0.051, 95% CI = −0.062, −0.039, *p* < 0.001) on cognitive function in the older adults was dissected into three pathways: (1) ACEs → Education level → Cognitive function (B = −0.015, 95% CI = −0.023, −0.008, *p* = 0.001); (2) ACEs → FOL → Cognitive function (B = −0.035, 95% CI = −0.044, −0.026, *p* < 0.001); and (3) ACEs → Education level → FOL → Cognitive function (B = −0.001, 95% CI = −0.001, 0.000, *p* = 0.012) (Supplementary Table S3). The effect of ACEs on cognitive function in the older population was partially serial mediated by educational level and FOL (Proportion mediated: 52.58%, see Supplementary Table S3). All models were adjusted age, gender, marital status, smoke, drink, rural living and education levels of both mothers and fathers.

## 4 Discussion

Building upon a large cohort study, this research delved into exploring the connection between ACEs and cognition. Additionally, it examined the extent to which education level and the FOL sequentially mediate this association. There are several noteworthy findings to highlight. First, ACEs were linked to diminished educational level, heightened FOL, and lower cognitive function. Furthermore, potential indirect pathways were identified involving the education and FOL between ACEs and cognitive function. In summary, our findings contribute to the existing literature by presenting new evidence supporting the role of educational level and FOL as mediators in the relationship between ACEs and cognition.

Consistent with previous research, our finding showed that ACEs were inversely related with education level. Giovanelli et al. discovered that individuals who experienced one or more conventional or expanded ACEs during early childhood were associated with fewer years of education, a diminished likelihood of achieving a bachelor’s degree or higher, and a decreased likelihood of obtaining an associate’s degree or higher. And they proposed that youths at higher risk can benefit most from early intervention for reducing ACE-related disparities (14). Numerous studies suggest that ACEs have significant impacts on individuals’ health and development, including affecting learning ability in school (32, 33), having a negative impact on children’s cognitive development (34), limiting educational resources and opportunities, and influencing individuals’ emotional regulation and coping strategies (35, 36). These effects collectively make it challenging for individuals to effectively deal with academic and life pressures. The cumulative impact of ACEs can create barriers to educational success and overall well-being.

Additionally, our examination also revealed that ACEs were positively associated with subjective FOL. Lin et al. found that individuals exposed to ACEs were at a higher risk of experiencing an increasing trajectory of FOL, with a dose–response relationship existing between the two (28). In the context of subjective experiences of social feelings, one review has highlighted the involvement of neurotransmitters, neurohormonal modulators, and specific brain regions in the mechanisms underlying loneliness (37). Furthermore, Brent and Silverstein et al. further proposed a link between early stress or adversity and the influence on oxytocin release and signaling (38). Oxytocin, known for its impact on interpersonal processes, affiliation, and feelings of love and trust, establishes a logical connection. This early influence may become deeply embedded in the life course, exerting enduring and long-term effects (39).

Emerging evidence showed that the long-lasting effect has been recognized that ACEs have a detrimental impact cognitive function in adults (31, 40). Prior researches investigated the association between ACEs and objective cognition (41, 42), which imply that adverse experiences in early life contribute to inferior cognitive outcomes as individuals age. One study, utilizing data from the English Longitudinal Study of Aging, demonstrated a subtle association between ACEs and memory decline over a ten-year period, spanning from middle to older age (42). Another study, which concentrated on older adults in rural South Africa, discovered a correlation between environmental ACEs, such as cohabiting with someone experiencing psychopathology (substance abuse or mental health challenges), and lower memory scores (43). There may be several potential mechanisms underlying the association between ACEs and cognitive impairment. Exposure to early-life adversity has crucial impact on brain development (44, 45). Specially, in animal models, there is direct evidence illustrating that stress during early life stages triggers structural, functional, and epigenetic alterations in brain regions linked to cognition. Notably, this includes a decrease in hippocampal volume, which has been associated with cognitive impairments, particularly memory deficits (46). These findings suggest that ACEs, education level and FOL should be taken into account as potential factors in measures of cognitive reserve, with the potential to reduce cognitive reserve.

The correlation between ACEs and cognition is mediated by both educational level and FOL. More precisely, individuals who experienced adverse events in their early lives were inclined to have a lower level of education, subsequently experiencing a heightened sense of FOL. This, in turn, elevated their likelihood of experiencing cognitive impairment. In general, both animal models and human studies have profoundly enriched our understanding of the neurodevelopmental mechanisms underlying the association between ACEs and the neural and behavioral phenotypes that result from a complex interplay between environmental, genetic, and epigenetic factors (47). Evidence indicated that early life adversity can impact life course development, including brain development, consequently leading to diminished cognitive, academic and behavioral performance and lower education attainment (6, 48). Moreover, individuals with lower levels of education in Latvia tend to experience higher levels of loneliness (49). This heightened loneliness is subsequently associated with lower scores in composite cognitive score, memory (immediate and delayed recall), verbal fluency, and backward digit span (50). Research findings highlight various measures of social isolation in long-term care settings and illuminate the contextual components that are correlated with a decrease in loneliness among older adults (51, 52). These measures may help to alleviate the cognition decline in older adults.

Our research possesses several limitations that should be acknowledged. Firstly, we did not thoroughly elucidate the relationship between the various components of ACEs and cognition. Secondly, the measures of ACEs exposure in our study utilized binary response options, which limited the exploration of adversity dimensions such as frequency, severity, duration, ages when it occurred, and the degree of psychological effects at the time. Employing more detailed and precise measures of ACEs may be essential to obtain accurate estimates of the relationships between ACEs and memory decline. Thirdly, the assessment of loneliness relied on a single item from ICSD-10. Future studies could enhance accuracy by utilizing structural questionnaires to evaluate the intricacies of FOL. For future research, to enhance the generalizability of findings and mitigate recall bias, incorporating diverse racial and cohort populations could be beneficial.

## 5 Conclusion

ACEs emerge as reliable predictors for educational level, the FOL, and cognitive function. Notably, chain mediation analyses revealed a novel underlying mechanism in the relationship between ACEs and cognitive function among the older population. ACEs serve as effective predictors for cognition by influencing educational level and the FOL. These findings are instrumental in early identification of individuals at risk for poor cognitive impairment, enabling interventions to exert the maximum impact on preventing or delaying cognitive and functional decline. Interventions aimed at optimizing cognitive health in the older adults and preventing future cognitive deficits should carefully consider the potential roles of ACEs, educational level, and the FOL within the relevant population.

## Data availability statement

The original contributions presented in the study are included in the article/Supplementary material. All data from CHARLS were accessible to the public through the website (http://charls.pku.edu.cn/).

## Ethics statement

This study was based on publicly available datasets. Ethical review and approval was not required for the study, in accordance with the local legislation and institutional requirements. The datasets are accessible for download on the CHARLS homepage at http://charls.pku.edu.cn/en. Approval for the CHARLS survey project was granted by the Biomedical Ethics Committee of Peking University, and all participants were required to provide informed consent through signing.

## Author contributions

XD: Writing – review & editing, Writing – original draft, Validation, Resources, Project administration, Methodology, Investigation, Formal analysis, Data curation, Conceptualization. MX: Writing – review & editing, Writing – original draft, Visualization, Validation, Resources, Project administration, Methodology, Investigation, Funding acquisition, Formal analysis, Data curation, Conceptualization. YW: Writing – original draft, Resources, Methodology, Investigation, Funding acquisition, Conceptualization. JC: Writing – original draft, Software, Methodology, Investigation, Funding acquisition, Data curation, Conceptualization. MZ: Writing – review & editing, Visualization, Validation, Supervision, Project administration, Methodology, Conceptualization. QW: Writing – review & editing, Validation, Supervision, Project administration, Methodology, Funding acquisition, Conceptualization.
