# Supplementary material for: Adverse childhood experiences and cognitive function in later life: the sequential mediating roles of education level and adult loneliness
Source: Front Public Health. 2024 Jul 16;12:1409966. doi: 10.3389/fpubh.2024.1409966 (PMC11288197; doi:10.3389/fpubh.2024.1409966)
Supplement: Supplementary file 1 [file Table_1.DOCX]

**Table S1 The distribution of educational attainment among both mothers and fathers.**

| **Education** | **Mother** | **Father** |
| --- | --- | --- |
| No formal education illiterate | 6471 (77.36%) | 3934 (47.03%) |
| Did not finish primary school but cap | 663 (7.93%) | 1279 (15.29%) |
| Sishu | 108 (1.29%) | 619 (7.40%) |
| Elementary school | 787 (9.41%) | 1442 (17.24%) |
| Middle school | 230 (2.75%) | 626 (7.48%) |
| High school | 64 (0.77%) | 230 (2.75%) |
| Vocational school | 31 (0.37%) | 132 (1.58%) |
| Two/Three Year College/Associate degree | 5 (0.06%) | 46 (0.55%) |
| Four Year College/Bachelor's degree | 6 (0.07%) | 55 (0.66%) |
| Post-graduated (Master/PhD) | 0 (0) | 1 (0.01%) |

**Table S2 Results of the chain mediating analyses. Estimate = non-standardized coefficient.**

|  | **Fit index** | | |  |  |  |  | **%95 CI** | |
| --- | --- | --- | --- | --- | --- | --- | --- | --- | --- |
|  | **R^2^** | **F** | **P** | **Estimate** | **SE** | **T** | **P** | **LCI** | **UCI** |
| **PATH: ACEs → Education level → FOL → Cognitive function** | | | | | | | | | |
| Dependent variable: **Education level** | | | | | | | | | |
| ACEs | 0.206 | 26.826 | < 0.001 | -0.058 | 0.016 | 3.526 | < 0.001 | -0.086 | -0.031 |
| Dependent variable: **FOL** | | | | | | | | | |
| ACEs | 0.031 | 7.786 | < 0.001 | 0.072 | 0.009 | 7.718 | < 0.001 | 0.057 | 0.087 |
| Education level |  |  |  | -0.022 | 0.006 | 3.917 | < 0.001 | -0.032 | -0.013 |
| Dependent variable: **Cognitive function** | | | | | | | | | |
| ACEs | 0.166 | 22.734 | < 0.001 | -0.047 | 0.031 | 1.489 | 0.136 | -0.096 | 0.005 |
| Education level |  |  |  | 0.261 | 0.021 | 12.328 | < 0.001 | 0.226 | 0.296 |
| FOL |  |  |  | -0.483 | 0.042 | 11.558 | < 0.001 | -0.551 | -0.416 |

Notes: ACEs: adverse childhood experiences; LCI: lower confidence interval; SE: standard error; UCL: upper confidence interval. The model adjusted age, gender, marital status, smoke, drink, rural living, and education levels of both mothers and fathers.

**Table S3 Results of the chain mediating effect based on Bootstrapping Test. Estimate = non-standardized coefficients.**

|  |  |  |  | **%95 CI** | |
| --- | --- | --- | --- | --- | --- |
|  | **Estimate** | **SE** | **P** | **LCI** | **UCI** |
| ACEs → Education level→ Cognitive function | -0.015 | 0.004 | 0.001 | -0.023 | -0.008 |
| ACEs → FOL → Cognitive function | -0.035 | 0.008 | < 0.001 | -0.044 | -0.026 |
| ACEs → Education attainment → FOL → Cognitive function | -0.001 | 0.000 | 0.012 | -0.001 | 0.000 |
| **Total mediation effects** | **-0.051** | **0.007** | **< 0.001** | **-0.062** | **-0.039** |
| Direct effect of ACEs on cognition | -0.047 | 0.031 | 0.136 | -0.097 | 0.005 |
| Total effects of ACEs on cognition | -0.097 | 0.032 | 0.002 | -0.150 | -0.045 |
| **Proportion mediated by mediators** | **52.58%** |  |  |  |  |

Notes: ACEs: adverse childhood experiences; FOL; feeling of lonely; LCI: lower confidence interval; SE: standard error; UCL: upper confidence interval. The model adjusted age, gender, marital status, smoke, drink, rural living, and education levels of both mothers and fathers.
